# Clinical Diagnosis of Nasal Discharges

**Published:** 1899-08

**Authors:** L. A. Merillat

**Affiliations:** Secretary of M’killip Veterinary College, Chicago


### DEPARTMENT OF SURGERY.

By L. A. Merillat, V.S.,
SECRETARY OF M'KILLIP VETERINARY COLLEGE, CHICAGO.

ASSISTED BY

W. E. A. Wyman, M.D.V., V.S.,
MILWAUKEE, WIS.

J. A. Sloan, B.Sc., M.D.V.,
SAN FRANCISCO, CAL.

Dr. Robert Jay, M.D.V.,
WEST LIBERTY, IA.

### Clinical Diagnosis of Nasal Discharges.

(Continued from page 444.)

Similarly as in nasal exhalations, the point of clinical interest of nasal discharges lies in their original site. In order to define it correctly the surgeon must submit it to a methodical and exhaustive macroscopical and at times microscopical examination.

The essential features in the various and variable nasal discharges are their odor, color, quantity, and consistency, and whether unilateral or bilateral.

*Odor.* While discussing nasal exhalations this point was fully covered, therefore the same necrotic states giving nasal exhalations a fetid odor also flavor nasal discharges. The odor of a discharge is of great clinical value. For instance, the peculiar odor of a gangrenous lung or the never-to-be-forgotten stink of a decaying tooth are diagnostic, while other discharges, no doubt equally noisome, nevertheless are not sufficiently peculiar in themselves to grant positive deductions; that most diseases are accompanied by an inodorous exudate is an interesting clinical fact.

*Color.* The color of nasal exudates is exceedingly variable, and changes even during the course of some diseases. It is readily seen that the color of nasal discharges depends on the amount and kind of diseased tissue present. At no time is the color a positive diagnostic agent, excepting possibly the orange, saffron, or rusty colored discharge seen during hemorrhagic hepatization of contagious pneumonia. It is to be admitted that a bloody discharge or one mixed with blood points distinctly toward certain lesions—thus, in chronic glanders intermittent limited hemorrhages are met with; disintegrating neoplasmata, injuries to the nasal mucous membrane, angioma, etc., are also accompanied by hemorrhages or exudates mixed with blood. Thus watery, gray, green, yellow, red, brown-colored exudates or shades and mixtures of the above are exhibited. At the same time a green discharge, as a rule, suggests nasal regurgitation of food-particles which contain chlorophyl, for instance, in pharyngitis; pus stained green by color-producing bacteria is also to be considered.

*Quantity.* The amount of nasal exudate depends on the nature and extent of the disease, which, appearing at one or both nostrils, becomes unilateral or bilateral. Unilateral nasal exudate is certainly a rare clinical sight compared with the bilateral form. Thus unilateral exudate means a diseased state in the nasal canal, cavity, sinus, etc., of that side, while a bilateral one may find its origin in the guttural pouches, pharynx, larynx, trachea, bronchi, ulcerative processes involving both nasal canals. Frequently the statement is supported that in glanders a unilateral discharge is diagnostic. Our experience decidedly opposes that view, as the exudate is as often bilateral as unilateral.

Clinically interesting is the fact that a sudden liberal flow following exercise, lowering of the head, manual pressure upon the guttural region suggests catarrhal states of the maxillary or frontal sinuses, guttural pouches, and the comparatively rare bronchiectasia. A limited discharge accompanies light nasal catarrhs, while a profuse exudate is met with in catarrh of the upper air-passages, as, for instance, in streptococcus distemper.

*Consistency.* The consistency of nasal discharges is also exceedingly variable; thus, in the first stage of an acute nasal catarrh it is watery, to become later of a sero-mucous or muco-purulent nature; the same is seen in influenza and simple catarrh of the upper air-passages. In chronic catarrh of the mucous membrane of the nose, in the second stage of acute coryza and catarrh of the upper air-passages, the exudate is more of a colorless, thin, watery, somewhat sticky, sero-mucous nature, or colorless, thick, stringy, quite sticky mucoid type. The muco-purulent exudate, slightly yellowish, gray, or greenish, is studied best in the streptococcus distemper. A nasal discharge of creamy consistency or, in other words, a purulent exudate is seen when abscesses open into the nasal cavity, bronchi, or pharynx (distemper). The consistency of nasal discharges in chronic catarrh of the sinuses, guttural pouches, is of a sero-mucoid type with an intermixture of fragments resembling curd. Of foreign admixtures in the exudate croupous membranes indicate diphtheritic processes of the larynx, trachea, or nasal canal. The foamy discharge contains air, as seen in pharyngitis, pharyngeal paralysis, or neoformations interfering with deglutition. Regurgitation of food or drink produces a variably colored discharge; thus, it may be yellowish (oats, straw), or green (grass, hay). The possibility of such food-particles coming from the stomach, while its source is not likely to be overlooked, nevertheless must be considered here.

After such an exhaustive clinical macroscopic survey the surgeon may find it agreeable to still further pursue the examination and test the exudate microscopically — glanders and distemper occupying the examiner's mind. In regard to glanders, the value of microscopical work with such a nasal exudate is one, on the whole, exceedingly doubtful, as the vast majority of veterinarians could not recognize them, on the principle that "all coons look alike to me," while the streptococcus of distemper is quite characteristic.

W. E. A. Wyman, M.D.V., V.S.

(To be continued.)

### Surgical Items.

A new method of tracheotomy consists of making a transverse incision between the cricoid cartilage and the first tracheal ring and the insertion of a flat, hook-shaped tube. A longitudinal incision is first made through the integument and muscles, and then a transverse one through the crico-trachealis ligament. This method is particularly recommended in young horses, because in these animals tracheal stenosis often results from the regular operation of cutting several tracheal rings.—L. A. M.

Any operation worth doing at all is worth doing well, and, therefore, the necessity of tying animals securely and specially for operations cannot be too often repeated. A running fight with a fistula, tumor, or quittor adds nothing to the credit of a surgeon.—*Id.*

Neurectomy of the larger nerve-trunks must be avoided in mules, as experience forcibly demonstrates that they are, for some unaccountable reason, very susceptible to trophic disturbances.—*Id.*

